# Interoception is Impaired in Children, But Not Adults, with Autism Spectrum Disorder

**DOI:** 10.1007/s10803-019-04079-w

**Published:** 2019-05-24

**Authors:** Toby Nicholson, David Williams, Katie Carpenter, Aimilia Kallitsounaki

**Affiliations:** School of Psychology, Keynes College, University of Kent, Canterbury, CT2 7NP UK

**Keywords:** Autism spectrum disorder, Interoception, Developmental disorders, Interoceptive accuracy

## Abstract

Interoception (the ability to sense what’s going on inside one’s body) is considered integral to many higher-order cognitive processes. Some have speculated that impaired interoception may underpin some features of ASD. Yet, in Experiment 1, we found no evidence of a between-group difference in either cardiac or respiratory interoceptive accuracy among 21 adults with ASD and 21 matched controls. Bayesian analyses suggested the data strongly supported the null hypothesis. In Experiment 2, we measured cardiac interoceptive accuracy in 21 *children* with ASD and 21 matched controls. Here interoceptve accuracy *was* significantly diminished in the ASD group and was associated with a moderate-to-large effect size. Results suggest early interoception difficulties are resolved or compensated for by adulthood in people with ASD.

**Electronic supplementary material:**

The online version of this article (10.1007/s10803-019-04079-w) contains supplementary material, which is available to authorized users.

## Introduction

Interoception refers to the sensation and representation of internal physiological signals, from organs such as the heart, stomach, lungs, and skin (Craig 2003). Its importance for survival is clear, but it is also increasingly seen as central to the development and organisation of higher-level cognition. For example, interoceptive accuracy (IA; the extent to which one can accurately identify interoceptive signals) has been found to be associated with ability in the domains of emotion-processing (Barrett et al. 2004; Pollatos et al. 2005), decision-making (Dunn et al. 2010), self-regulation (Herbert et al. 2007), empathy (Fukushima et al. 2011) and theory of mind (Shah et al. 2017). These wide ranging associations are in line with theories of embodied cognition that imply cognition is situated in bodily systems, and also point towards a role for interoception in both self and other processing (Gallese 2007; Barrett and Simmons 2015; Barrett 2017). This has led to a growing interest in the importance of interoception for disorders such as autism spectrum disorder (ASD).

ASD is a neurodevelopmental disorder which is characterised by severe behavioural impairments in social-communication and behavioural flexibility (American Psychiatric Association 2013). At the cognitive level, ASD is characterised by a difficulty with representing others’ mental states (theory of mind; Yirmiya et al. 1998) and with one’s own mental states (metacognition; e.g., Brosnan et al. 2016; Grainger et al. 2014; McMahon et al. 2016). Given recent findings linking interoception with higher level cognitive processes, such as theory of mind (Shah et al. 2017) and self-regulation (Herbert et al. 2007), it has been argued that difficulties with self-other processing in ASD may be the result of an underlying impairment in interoceptive awareness. Quattrocki and Friston (2014) suggest that abnormal regulation of the oxytocin (a hormone linked to social bonding) system in ASD may represent the biological basis of interoceptive impairments, which in turn result in problems linking more abstract concepts associated with these bodily signals. The idea is that a difficulty interpreting one’s own internal bodily signals early on may interfere with learning about the association between these low level bodily signals and other higher level feelings and thoughts, restricting the comprehension of oneself, which in turn would have similar effects on understanding other selves. However, empirical studies of interoception in ASD have produced decidedly mixed results.

The majority of studies assessing interoception in ASD have used the heartbeat tracking task (Schandry 1981). In this task participants are asked to count the number of times they feel their heart beat in a given time interval without touching their body (i.e., feeling their pulse). The closer the participant’s reported number of heart beats is to their actual number of heart beats, the greater their interoceptive accuracy (IA). Six studies have used the heartbeat tracking task to measure interoceptive accuracy among individuals with ASD and findings are summarised in Table 1. In short, while diminished accuracy was observed in two studies, four studies found only negligible and non-significant between-group differences suggesting that interoceptive accuracy is not impaired in ASD. One plausible explanation for the mixed findings is proposed by the alexithymia hypothesis (Bird and Cook 2013), which posits that difficulties processing emotions are only evident in ASD individuals who also have high levels of alexithymia (defined as the inability to identify or describe ones emotions; Taylor 1984). According to this view interoceptive abnormalities are a central component of alexithymia, not autism, and the suggested link between ASD and interoception can be explained by the high comorbidity of ASD and alexithymia (approximately 50% of ASD individuals also have alexithymia, five times that of the general public; Berthoz and Hill 2005), as opposed to ASD itself. Therefore, mixed results in the literature might emerge because levels of alexithymia vary from study to study, with only studies including ASD individuals with high levels of alexithymia likely to show a difference in interoception (Shah et al. 2016).

**Table 1:** Details of previous studies of interoceptive accuracy in ASD

Study	Participant characteristics	Key IA findings	Control task?	Alexithymia
Schauder et al. (2015)	*Children* 21 ASD, (M age: 12.3, SD 2.8), 24 NT (M: 11.5, SD 2.5), matched for age and IQ	ASD = NT No group difference	Visual counting task: a subset of children, 8 TD (33%) and 12 ASD (57%), counted dim visual stimulus over same interoception time intervals. Performance did not differ between groups	Not measured
Shah et al. (2016)	*Adults* 19 ASD (M: 32.9, SD 11.5) 19 NT, (M: 32.9, SD: 14.4), matched for age, gender and IQ	ASD = NT No group difference	Time estimation task: All participants were instructed to judge the duration of three randomized intervals. No correlation between time estimation and IA	Negative correlation between Alexithymia and IA in NT (exp1) and ASD and NT (exp2)
Garfinkel et al. (2016b)	*Adults* 20 ASD (M 28.1, SD 8.8), 20 NT (M 27.8, SD 3.4), matched for age and gender, but IQ not measured	**ASD < NT (d = 1.10)** **ASD significantly lower IA**	None	Not measured
Mash et al. (2017)	*Children* 52 ASD (M 12.2, SD 2.9) 42 NT, (M 11.03, SD 2.8) and *Adults* 19 ASD (M: SD), 19 NT (M: SD:), matched for age and gender, significantly differ on IQ, both across whole sample and within age groups	ASD = NT When groups split by IQ (over or under 115) for the groups under 115 the NT’s showed positive correlation between age and IA (r = 0.539) while ASD showed negative correlation (r = − 0.339)	Visual counting task: all adults, and 28 ASD (54%) and 21 NT (50%) children counted frequency of visual stimulus. NT significantly better (p = 0.004) and significant correlation between IA and task for NT’s only (r = 0.35)	Not measured
Palser et al. (2018)	*Children* 30 ASD, (M: 12.5, SD 2.9), 30 NT (M: 11.9, SD 3), matched for age, VIQ, PIQ, and FSIQ	**ASD < NT (d = 1.09)** **ASD significantly lower IA**	Auditory counting task: 12 ASD (40%) and 17 NT (57%) participants had to silently count auditory-presented tones that numbered 5, 20 or 33. No group differences on task. No other comparisons reported	Not measured
Nicholson et al. (2018)	*Adults* 46 ASD (M:40.2 SD 11.7), 48 NT (M: 41.2, SD 12.6), matched for age, sex, VIQ, PIQ, and FSIQ	ASD = NT No group difference	None	ASD Hi (> 60), n = 27, and low (< 60), n = 19, in Alexithymia did not differ in IA; No correlation between IA and Alexithymia

A recent study tested this hypothesis directly comparing a group of autistic adults who manifested clinically significant levels of alexithymia (n = 27) with an age, sex, IQ, and ASD severity-matched group of autistic adults who did not meet criteria for alexithymia (n = 19) (Nicholson et al. 2018). Crucially, there was no hint of any difference between these two sub-groups of ASD participants in terms of interoceptive accuracy, which provided clear evidence against the alexithymia hypothesis. One of the potential limitations of this recent study, however, was the lack of a control task to guard against alternative explanations for the ASD group’s undiminished performance on the heartbeat tracking task. One potential difficulty with the heartbeat tracking task is that it might be possible to achieve apparently good interoceptive accuracy on it by counting silently. Given that the average heart rate is around 60 bpm, simply counting seconds internally could be sufficient to achieve high interoceptive accuracy even if one was unable to detect one’s heartbeat (Brener and Ring 2016). Therefore, in the current study we adopted a version of a time estimation control task (employed by Shah et al. 2016), which required participants to count silently during several unspecified periods and then estimate how many seconds had passed during the time. If participants with ASD (or neurotypical comparison participants) were compensating for underlying difficulties with interoception by silently counting, then performance on the time estimation control task should be associated significantly with performance on the heartbeat tracking task and group differences on the latter should appear once time estimation is controlled for.

A second more general limitation with the field as a whole concerns the reliance on the heartbeat tracking task as a single measure of interoceptive accuracy. The task is used frequently because it is relatively easy to administer, has good test–retest reliability (Mussgay et al. 1999), and is sensitive to individual differences (Christensen et al. 2018; Dunn et al. 2010; Garfinkel et al. 2015). However, some have questioned whether interoception is a unitary construct and suggested that it actually involves multiple independent systems, which may not be causally linked (Garfinkel et al. 2016a). If this latter view is correct, then it may be that individuals with ASD show diminished interoceptive accuracy on one domain, but not another domain. Experiment 1 addresses these issues.

In Experiment 1, we gave 21 adults with ASD and 21 NT age- and IQ-matched comparison adults the classic heartbeat tracking task to measure cardiac interoception, as well as a version of a task used by Murphy et al. (2018) to test respiratory interoceptive accuracy. We included control tasks designed to assess the extent to which alternative, compensatory strategies might be used by individuals with ASD to succeed on the two interoception tasks in the absence of underlying interoceptive competence. We also administered measures of mindreading, depression and anxiety to allow full characterisation of the sample and for the exploration of underlying cognitive/personality correlates of interoceptive accuracy. We predicted that adults with ASD would be unimpaired on both the cardiac and respiratory interoception tasks and that this would reflect truly undiminished interoceptive accuracy (rather than compensatory strategy use).

## Experiment 1

### Method

#### Participants

The experiment was ethically approved by the School of Psychology Research Ethics Committee at the University of Kent (Ref: 201815259101245011). Twenty-one adults with ASD (8 female) and 21 NT comparison adults (6 females) took part in Experiment 1. Participants with ASD had an average age of 37.24 (SD 11.85; range 24–64 years) and NT participants had an average age of 41.19 (SD 14.02; range 24–65 years). Groups were matched in relation to verbal, performance and full-scale IQ (using the Wechsler Abbreviated Scale for Intelligence-II; Wechsler 1999), as well as age (see Table 2). All participants also completed the Autism-spectrum Quotient (AQ; Baron-Cohen et al. 2001b), and two mindreading tasks; the reading the mind in the eyes task (RMIE; Baron-Cohen et al. 2001a) and the animations task (Abell et al. 2000).

**Table 2:** Experiment 1 participant characteristics, matching statistics and secondary measures

	ASD (*n* = 21)	NT (*n *= 21)	*t*	*p*	*d*	BF_10_
Age	37.24 (11.85)	41.19 (14.02)	0.99	0.33	0.30	0.45
VIQ	105.52 (11.44)	104.29 (10.99)	0.36	0.72	0.11	0.32
PIQ	105.71 (17.62)	105.14 (14.95)	0.11	0.91	0.03	0.31
FSIQ	106.00 (13.51)	105.52 (12.67)	0.12	0.91	0.04	0.31
TAS	61.52 (11.75)	44.33 (9.91)	5.13	< 0.001	1.58	> 100
AQ	33.00 (8.20)	14.95 (5.49)	8.38	< 0.001	2.59	> 100
ADOS	9.29 (4.80)	–	–	–	–	–
RMIE (prop. accuracy)	0.70 (0.16)	0.77 (0.10)	1.77	0.04	0.52	1.95
Animations (prop. accuracy)	0.57 (0.31)	0.71 (0.19)	1.82	0.04	0.54	2.11
BMI	26.17 (5.87)	26.13 (4.57)	0.26	0.98	< 0.01	0.30
STAI	95.62 (21.02)	72.29 (19.11)	3.77	0.001	1.16	52.29
BDI	15.33 (11.91)	9.14 (7.89)	1.99	0.05	0.61	1.42

*VIQ* verbal IQ, *PIQ* performance IQ, *FSIQ* full scale IQ, *TAS* Toronto alexithymia scale, *AQ* autism quotient, *ADOS* autism diagnostic observation schedule, *RMIE* reading the mind in the eyes, *BMI* body mass index, *STAI* state/trait anxiety inventory, *BDI* Beck Depression Inventory 2

The AQ (Baron-Cohen et al. 2001c) is a widely used self-report questionnaire that measures reliably ASD traits, in both general and clinical populations (e.g., Reed et al. 2011; Williams et al. 2018). In this questionnaire, individuals are asked to indicate the extent to which they agree with each of the 50 statements (e.g., “I find social situations easy”) that the questionnaire comprises, using a 4-point Likert scale, ranging from “definitely agree” to “definitely disagree”. Scores range from 0 to 50, with higher scores indicating more ASD traits. A score of ≥ 26 is the cut-off point that denotes clinically significant levels of autism traits (Woodbury-Smith et al. 2005).

The RMIE (Baron-Cohen et al. 2001c) is a reliable and widely used measure of mindreading abilities in general and clinical populations (e.g., Domes et al. 2007). Participants are presented with a series of 36 photographs showing the eye-region of males and females, and they are asked to choose among four different options the emotion/feeling that best describes the mental state of the depicted person. Scores range from 0 to 36, with higher scores indicating better mindreading abilities.

The Animations task, which is based on Heider and Simmel (1944), required participants to describe interactions between a large red triangle and a small blue triangle, as portrayed in a series of silent video clips (Abell et al. 2000). Four clips were apt to invoke an explanation of the triangles’ behaviour in terms of epistemic mental states, such as belief, intention, and deception. These clips comprise the “mentalizing” condition of the task and were employed in this experiment. Each clip was presented to participants on a computer screen. After the clip was finished, participants described what had happened in the clip. An audio recording of participants’ responses was made for later transcription. Each transcription was scored on a scale of 0–2 for accuracy (including reference to specific mental states), based on the criteria outlined in Abell et al. (2000). Eighty percent of transcripts were also scored by an independent rater. Inter-rater reliability was excellent according to Cicchetti’s (1994) criteria (intra-class correlation = 0.86). Accuracy (proportion) among ASD and comparison participants is shown in Table 2.

Participants in the ASD group had received verified diagnoses in line with standard criteria (APA 2000; World Health Organisation 1993). Diagnostic reports/letters were validated by researchers prior to testing. The Autism Diagnostic Observation Schedule (ADOS; Lord et al. 2000), an in depth observational measure of ASD characteristics, was also administered by a research-reliable assessor to all members of the ASD group. The ADOS and AQ were employed as indices of the severity of ASD features, and all participants met the relevant cutoff score on at least one of these measures. Six of the 21 participants with ASD did not score above the ASD cut-off on the ADOS (with scores of 6, 6, 4, 4, 2, and 0, respectively). However, the ADOS does not have perfect sensitivity to ASD (for example, see Risi et al. 2006) and a sub-threshold score does not over-rule a formal diagnosis. Thus, excluding participants on the basis of a sub-threshold ADOS score would result in an unrepresentative sample of participants. Most important, when we excluded participants with ASD who scored under the ADOS cutoff of seven, the between-group differences in experimental task performance remained almost identical in substance; that is, all p values that were significant when these six participants were in included remained significant when they were excluded, all p values that were non-significant remained non-significant, and all effect sizes remained in the same order of magnitude (small/medium/large). Therefore, results from all 21 participants are reported in the “Results” section below.

### Materials and Procedures

#### Interoception Tasks

##### Heartbeat Tracking Task

In the heartbeat tracking task participants index finger was attached to a pulse oximeter (Contec Systems CMS-50D+; Qinhuangdao, China) which measured their heart rate. During the task they were asked to close their eyes and silently count how many times they felt their heart beat in between two auditory tones. This procedure was repeated over four different time intervals (25, 35, 45 and 100 s) and presented in a random order. Cardiac Interoceptive Accuracy (cIA) was calculated as: 1—(|recorded number of heartbeats—counted number of heartbeats|)/((recorded heartbeats + counted number of heartbeats)/2), (Garfinkel et al. 2015). This provided a value between − 1 and 1 for each time interval, with more positive values indicating better cIA. The materials and procedures for the heartbeat tracking task were the same as used in previous studies of cIA in ASD (Schauder et al. 2015; Shah et al. 2016; Mash et al. 2017; Nicholson et al. 2018).

##### Blow Comparison Task

In the blow comparison task, participants exhaled into a peak flow meter (Mini-Wright) at a particular level of effort (a “target blow”) and then attempted to reproduce exactly the intensity of the target blow in a second exhalation 15 s later (a “comparator blow”). Successful reproduction of the target blow required participants to monitor and perceive the feeling of their target blow as they were engaged in producing it, with the aim of remembering and recreating that feeling when producing the comparator blow. Therefore, in order to perform the task one needed to encode the bodily effort/exertion during the target exhalation and remember that feeling in order to recreate it, both of which rely on interoceptive information relating to the feeling of ones’ breath.

Participants completed a total of 9 target blows (and 9 corresponding comparator blows), three blows at each of three intensities (weak, medium, and firm). Prior to beginning a practice the experimenter provided the participant with an example of each of the three intensities by demonstrating each themselves. For example, for the weak intensity trials, participants were instructed to “blow into the peak flow meter with a weak intensity, like this. Now you try.” After demonstrating each intensity level, the experimenter checked that the participant could feel the difference between the levels before beginning the practice, which included the participant trying each intensity themselves. On each trial, the measurement of the target exhalation was hidden from view during the task so that participants could only rely on their internal feeling of exhaling to complete the subsequent comparator blow. Trials at each intensity were blocked together, such that all weak trials were performed sequentially, all medium trials were performed sequentially etc. Order of trial intensity was counterbalanced across participants. Trials were blocked according to effort intensity to avoid an overly high burden on long-term memory for the target blow. Had trials not been blocked by intensity, a participant might have, for example, given a firm target blow, followed immediately by an equivalent comparator blow, but then not produced their second or third firm comparator blows for several minutes. The reliance on memory for one’s firm target blow from minutes before would have risked turning the task into a test of long-term memory as much as, or more than, a test of interoception. By blocking trials according to intensity level, participants should have been able to maintain awareness of internal effort in working memory, thus making the task a valid measure of interoception.

Between the different intensity blocks a 40 s break was given to participants to allow their breathing to return to normal. Respiratory interoceptive Accuracy (rIA) was calculated in the same manner as the cIA: 1 − (target blow − comparator blow)/((target blow + comparator blow)/2).

#### Control Tasks

##### Time Estimation

Following the same procedure as in the heartbeat tracking task, participants were asked to count the number of seconds of three different time intervals (19, 37, and 49 s), with the order of intervals being randomised across participants. Accuracy was quantified using the same formula used to measure cardiac interoceptive accuracy. If performance on the heartbeat tracking task is the result of time estimation rather than interoception then one would predict a positive correlation between the two measures. If, however, no such correlation exists it supports the idea that participants are not merely counting during the heartbeat tracking task. Moreover, time estimation task performance can be covaried with the heartbeat tracking task in order to assess the extent to which performance on the task can explain performance on the heartbeat tracking task and how this differs between groups.

##### Memory Task

Between-group differences in rIA could be explained by between-group differences in short-term memory for internal effort. Therefore, we employed a memory control task with a parallel structure to the blow comparison task in order to compare performance on the two tasks and to ensure that short-term memory could not explain rIA performance. In the memory task participants were asked to press the “b” key of a keyboard down, producing a tone and then release the key whenever they wanted to (with the caveat that it must be for more than a second so that participants could not merely touch the key very briefly, therefore potentially making the comparison press easier). Subsequently, they were asked to repeat the task with the aim of pressing the button down for the same length of time, as they previously did. They were instructed to rely solely on their memory to recreate the duration of the prior tone and not to count seconds. Participants completed one practice trial and then nine experimental ones. Again accuracy was measured using the same formula as the interoception and time estimation tasks. If performance on the blow comparison task is the merely the result of short-term memory for external cues, then performance on the blow comparison task should positively correlate with performance on the memory task.

#### Self-report Measures

In addition to the experimental interoception tasks and mindreading tasks we also included a series of self-report measures in order to assess levels of depression (Beck Depression Inventory 2; Beck et al. 1996), anxiety (State-trait Anxiety Inventory; Spielberger et al. 1983), and alexithymia (Toronto alexithymia scale; Taylor 1984) in our groups. Additionally, we also calculated each participant’s body mass index. Each of these measures is described in the Online Supplementary Material. Including these measures allowed the chance to explore the association between each of them and interoceptive accuracy on the experimental tasks. However, this was not the primary purpose of the study and, to retain the focus on between-group effects, we report the results from these correlations in the Supplementary Material.

##### Statistical Analyses

In addition to null hypothesis significance testing, we also conducted Bayesian analyses to provide an estimation of the relative strength of findings for one hypothesis over another (i.e., the alternative hypothesis over the null, or vice versa). This allows a more graded interpretation of the data than is possible using P values or effect sizes alone (e.g., Dienes 2014; Rouder et al. 2009). According to Jeffreys’ (1961) criteria, Bayes factors (BF10) > 3 provide firm evidence for the alternative hypothesis (with values > 10, > 30, and > 100 providing strong, very strong, and decisive evidence, respectively) and values under 1 provide evidence for the null (with values < 0.33 providing firm evidence). BF^10^ values can be considered to reflect the likelihood that the alternative hypothesis is more likely to be true than the null hypothesis. Hence, a BF^10^ of 3 suggests the alternative hypothesis is three times more likely to be true than the null hypothesis. Bayesian analyses were conducted using JASP 0.8.1 (JASP Team 2016).

### Results

#### Interoception Task 1: Heartbeat Tracking Task

**Fig. 1:**
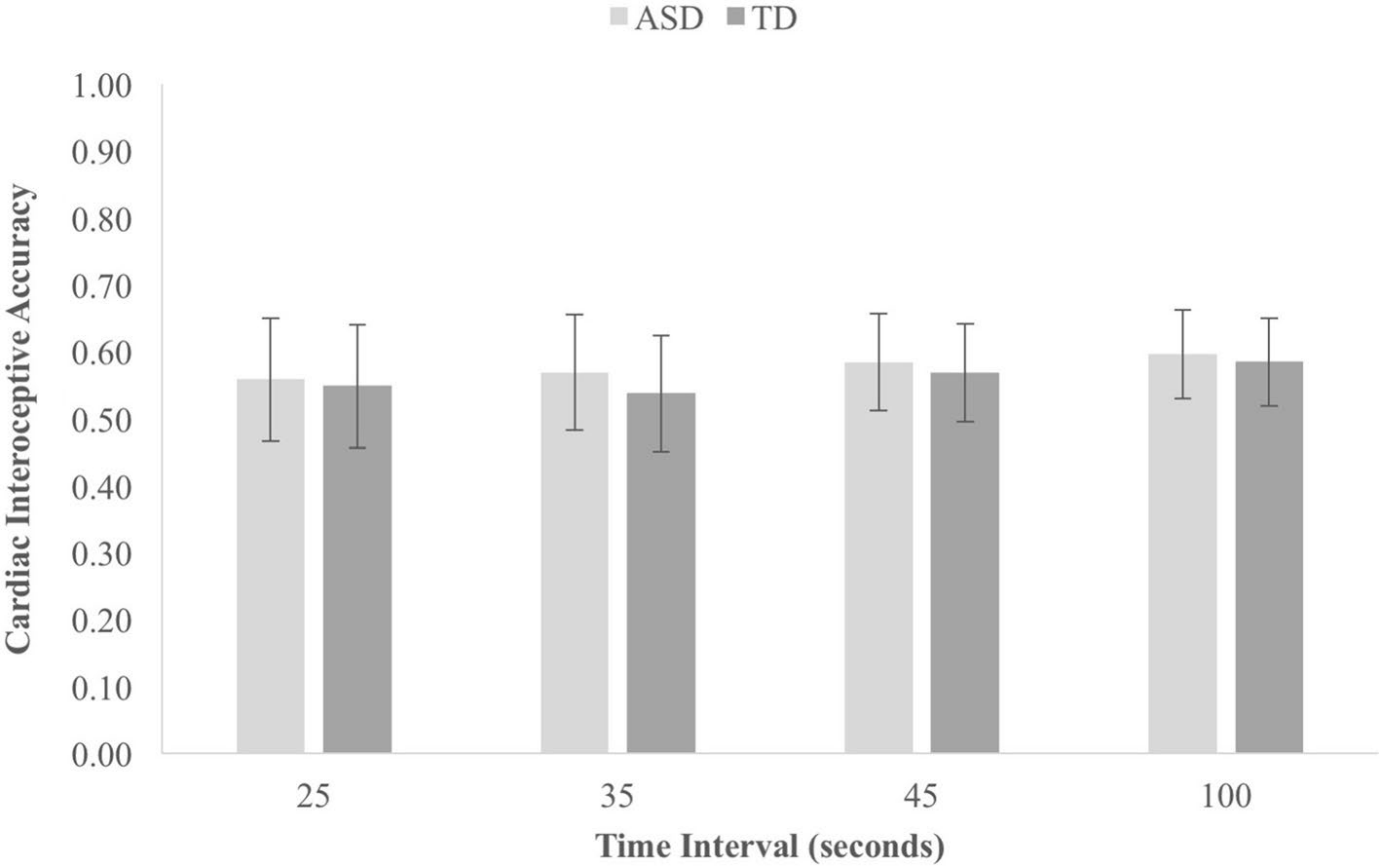
Cardiac interoceptive accuracy scores for each time period for each group during the HTT in experiment one. Error bars represent SEM

The average cIA score was 0.58 (SD 0.32) in the ASD group and 0.56 (SD 0.30) in the NT group, a difference which was statistically small and nonsignificant, *t*(40) = 0.18, *p* = 0.86, *d* = 0.06, BF^10^ = 0.31. The mean cIA score for each time interval on the HTT in each group is shown in Fig. 1. A 2 (group: ASD/NT) × 4 (time interval: 25 s/35 s/45 s/100 s) ANOVA indicated a non-significant main effect of Group, *F*(1,40) = 0.32, *p* = 0.86, $\eta_{p}^{2} = 0.001$, and a nonsignificant Group × Time Interval ienteraction ffect, *F*(3,120) = 0.02, *p* = 0.99, $\eta_{p}^{2} = 0.001$. Therefore, in line with most other studies of IA in ASD, there was no significant difference between groups in levels or patterns of performance on the heartbeat tracking task.

To assess the impact of levels of alexithymia on cardiac interoception we split the ASD group into those over (n = 13) the threshold for the diagnosis of alexithymia (> 60 on TAS20) with those under (n = 8) the threshold (< 60). The average cIA score was 0.60 (SD 0.16) in the under threshold group and 0.56 (0.40) in the over threshold group, a difference which was statistically small and nonsignificant, *t*(19) = 0.26, *p* = 0.80, *d* = 0.13, BF^10^ = 0.41. Therefore the presence or absence of alexithymia could not explain ASD performance on the heartbeat tracking task.

#### Time Estimation Control

The average time estimation score was 0.80 (SD 0.20) in the ASD group and 0.74 (SD 0.17) in the NT group, a difference which was non-significant, *t*(40) = 1.01, *p* = 0.32, *d* = 0.31, BF^10^ = 0.46. Performance on the time estimation task was non-significantly associated with the heartbeat tracking task in either the ASD group, *r* = -0.02, *p* = 0.92, BF^10^ = 0.27, or the NT group, *r* = 0.19, *p* = 0.42, BF^10^ = 0.37. Therefore, in neither group did it appear that performance on the heartbeat tracking task reflected merely time estimation ability. Therefore, it is unlikely that the null group finding can be explained by use of alternative, compensatory strategy use among participants with ASD. Indeed, after controlling for performance on the time estimation task, the main effect of Group on heartbeat tracking task performance remained small and non-significant in an ANCOVA, *F*(2,39) = 0.11, *p *= 0.92, $\eta_{p}^{2} < 0.00 1$.

#### Interoception Task 2: Blow Comparison Task

**Fig. 2:**
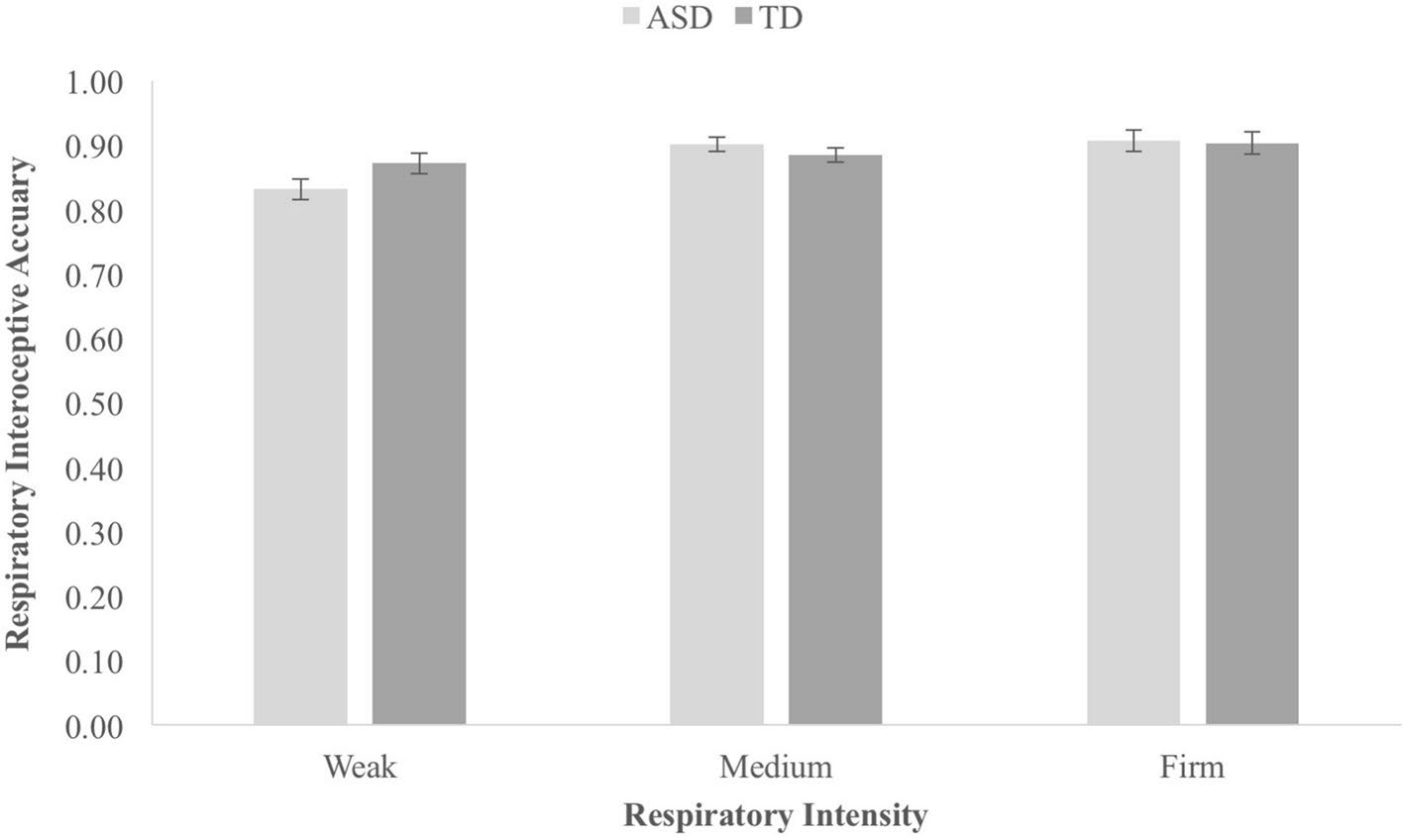
Respiratory interoceptive accuracy scores for each level of intensity for each group during the BCT in experiment one. Error bars represent SEM

The average rIA score was 0.88 (SD 0.04) in the ASD group and 0.89 (SD 0.04) in the NT group, a difference which was statistically small and nonsignificant, *t*(40) = 0.50, *p* = 0.62, *d* = 0.15, BF^10^ = 0.33. The mean rIA score for each intensity on the blow comparison task for each group is shown in Fig. 2. A 2 (group: ASD/NT) × 3 (intensity: weak/medium/firm) ANOVA was conducted. Neither the main effect of Group, *F*(2,40) = 0.29, *p* = 0.59, $\eta_{p}^{2} = 0.00 7$, nor the Group × Intensity interaction effect, *F*(2,80) = 2.20, *p* = 0.18, $\eta_{p}^{2} = 0.0 5 2$, was significant. Therefore, there was no significant difference between groups in either levels or patterns of performance on the blow comparison task.

Once again to assess the impact of levels of alexithymia on respiratory interoception we split the ASD group into those over (n = 13) the threshold for the diagnosis of alexithymia (> 60 on TAS20) with those under (n = 8) the threshold (< 60). The average rIA score was 0.88 (SD 0.06) in the under threshold group and 0.88 (0.04) in the over threshold group, a difference which was statistically small and nonsignificant, *t*(19) = 0.13, *p* = 0.90, *d* <0.01, BF^10^ = 0.40. Therefore, the presence or absence of alexithymia could not explain ASD performance on the blow comparison task.

#### Memory Control Task

Due to experimenter error, one NT participant failed to complete the memory task. The average memory task score was 0.85 (SD 0.07) in the ASD group and 0.87 (SD 0.09) in the NT group, a difference which was small and non-significant, *t*(39) = 0.57, *p* = 0.57, *d* = 0.18, BF^10^ = 0.31. Performance on the memory task was non-significantly associated with the blow comparison task in either the ASD group, *r* = 0.17, *p* = 0.47, BF^10^ = 0.35, or the NT group, *r* = 0.23, *p* = 0.32, BF^10^ = 0.44. Therefore, it is unlikely that the null group finding can be explained by use of alternative, compensatory strategy use among participants with ASD. Indeed, after controlling for performance on the memory control task, the main effect of Group on blow comparison task performance remained small and non-significant in an ANCOVA, *F*(2,38) = 0.13, *p *= 0.73, $\eta_{p}^{2} = 0.00 3$.

#### Interoceptive Measures

cIA was nonsignificantly correlated with rIA in both the ASD group, *r* = 0.06, *p* = 0.80, BF^10^ = 0.28, and the NT group, *r* = 0.17, *p* = 0.46, BF^10^ = 0.35, suggesting that interoception related to the heart and interoception related to lungs are independent processes and not mediated by a unitary system.

#### Secondary Measures

Figures concerning the comparison between groups on the secondary measures can be found in Table 2.

#### Association Analyses

A series of bivariate and partial correlations were conducted to explore the association between interoceptive domains, ASD traits/features and the other secondary measures. Given that the results from association analyses were not a central focus the current study, combined with the fact that none of the key correlations was significant, means we report the data in Online Supplementary Material.

### Experiment 1: Discussion

The results of Experiment 1 support previous research by Nicholson et al. (2018) and others that indicates no differences between autistic and non-autistic adults in cardiac IA (Schauder et al. 2015; Shah et al. 2016; Nicholson et al. 2018). Importantly, Bayesian analyses consistently suggested that the data strongly supported the null hypothesis. We confirmed in the current experiment that such a lack of significant between-group difference in cIA is unlikely to be the result of unmeasured confounds or alternative strategy use. Rather, cardiac interoceptive accuracy appears to be a relative strength for autistic adults, in line with theories that suggest awareness of own physiological states is undiminished in ASD (e.g., Grainger et al. 2016; Lind 2010; Uddin 2011; Williams et al. 2018). To confirm that this strength was not only in the cardiac domain, we also employed a novel measure of respiratory interoception and, again, failed to observe a between-group difference in interoceptive accuracy.

These findings add to the growing literature investigating interoception in ASD, and challenges theories claiming interoceptive impairments lie at the heart of the disorder (Quattrocki and Friston 2014). One possibility that might save this latter theory and explain contradictory findings in the literature is if interoception impairments are apparent in children with ASD, even if not adults with ASD. As Williams and Bowler (2014, p. 5) note,

“we should never forget that the clinical picture we see among individuals with a diagnosis of ASD represents a particular point in an atypical developmental trajectory, in which both the clinical features and any putative underlying factors may be in a process of change.”

It may be that early impairments in interoception resolve, or are compensated for, by the time autistic individuals reach adulthood. Of the three studies looking at cardiac interoception in *children* with ASD, two found no between-group differences in cIA (Schauder et al. 2015; Mash et al. 2017). However, the most recent study, by Palser et al. (2018), found a large interoceptive impairment in children with ASD, relative to comparison children. Therefore, in Experiment 2 we ran the same cIA task in children with and without ASD in order to add to the existing studies investigating interoception in ASD, and to test the hypothesis that any impairment associated with the condition may be developmental in nature and therefore more pronounced in childhood. Based on the findings from Experiment 1 and our own theoretical inclinations, we predicted that children with ASD would be unimpaired on the heartbeat tracking task and that this would reflect truly undiminished interoceptive accuracy (rather than compensatory strategy use).

## Experiment 2

### Method

#### Participants

21 children with ASD (5 female) and 21 NT comparison children (6 females) were recruited to take part. Participants with ASD had an average age of 12.95 (SD 1.49; range 10–16 years) and NT participants had an average age of 12.70 (SD 1.17; range 24–65 years). Groups were matched in relation to verbal, performance and full scale intelligence, as measured by the Wechsler Abbreviated Scale for Intelligence-II (Wechsler 1999), as well as age. Participants completed the same two mindreading tasks used in Experiment 1 (see Table 3). Firstly, participants completed a child version of the RMIE (Baron-Cohen et al. 2001c). The child version of the reading the mind in the eyes included 28 photographs from the adult version of the task with the four word options representing simplified more basic language. Secondly, participants did the same animations described in Experiment 1. For the animations task all transcripts were also scored by an independent rater. Inter-rater reliability was excellent according to Cicchetti’s (1994) criteria (intra-class correlation = 0.85). For both mindreading tasks proportion scores were used to allow easy comparison with experiment one. Participants in the ASD group had received verified diagnoses in line with standard criteria (APA 2000; World Health Organisation 1993). The Social Responsiveness Scale was also completed for each participant by a parent or caregiver.

**Table 3:** Experiment 2 participant characteristics and matching statistics

	ASD (*n* = 21)	NT (*n *= 21)	*t*	*p*	*d*	BF_10_
Age	12.95 (1.49)	12.70 (1.17)	0.60	0.55	0.19	0.35
VIQ	107.95 (9.11)	108.71 (11.22)	0.24	0.81	0.07	0.31
PIQ	105.71 (17.62)	105.14 (14.95)	0.16	0.87	0.03	0.31
FSIQ	111.76 (14.90)	112.48 (14.13)	0.20	0.85	0.05	0.31
SRS	83.90 (10.02)	46.38 (11.26)	11.41	< 0.001	3.52	> 100
RMIE (prop. correct)	0.70 (0.08)	0.72 (0.08)	0.71	0.48	0.25	0.37
Animations (prop. correct)	0.44 (0.23)	0.71 (0.17)	4.40	< 0.001	1.34	>100

*VIQ* verbal IQ, *PIQ* performance IQ, *FSIQ* full scale IQ, *SRS* social responsiveness scale, *RMIE* reading the mind in the eyes

### Materials and Procedures

Participants from each group completed the same heartbeat tracking task and time estimation tasks used in Experiment 1. We did not include the Blow Comparison Task in Experiment 2, because we had limited time with the children in Experiment 2 to complete testing (unlike with the adults in Experiment 1). Therefore, and made the strategic decision to include only the heartbeat tracking task, because of its historical use in the literature and familiarity with researchers.

### Experiment 2: Results

#### Heartbeat Tracking Task

**Fig. 3:**
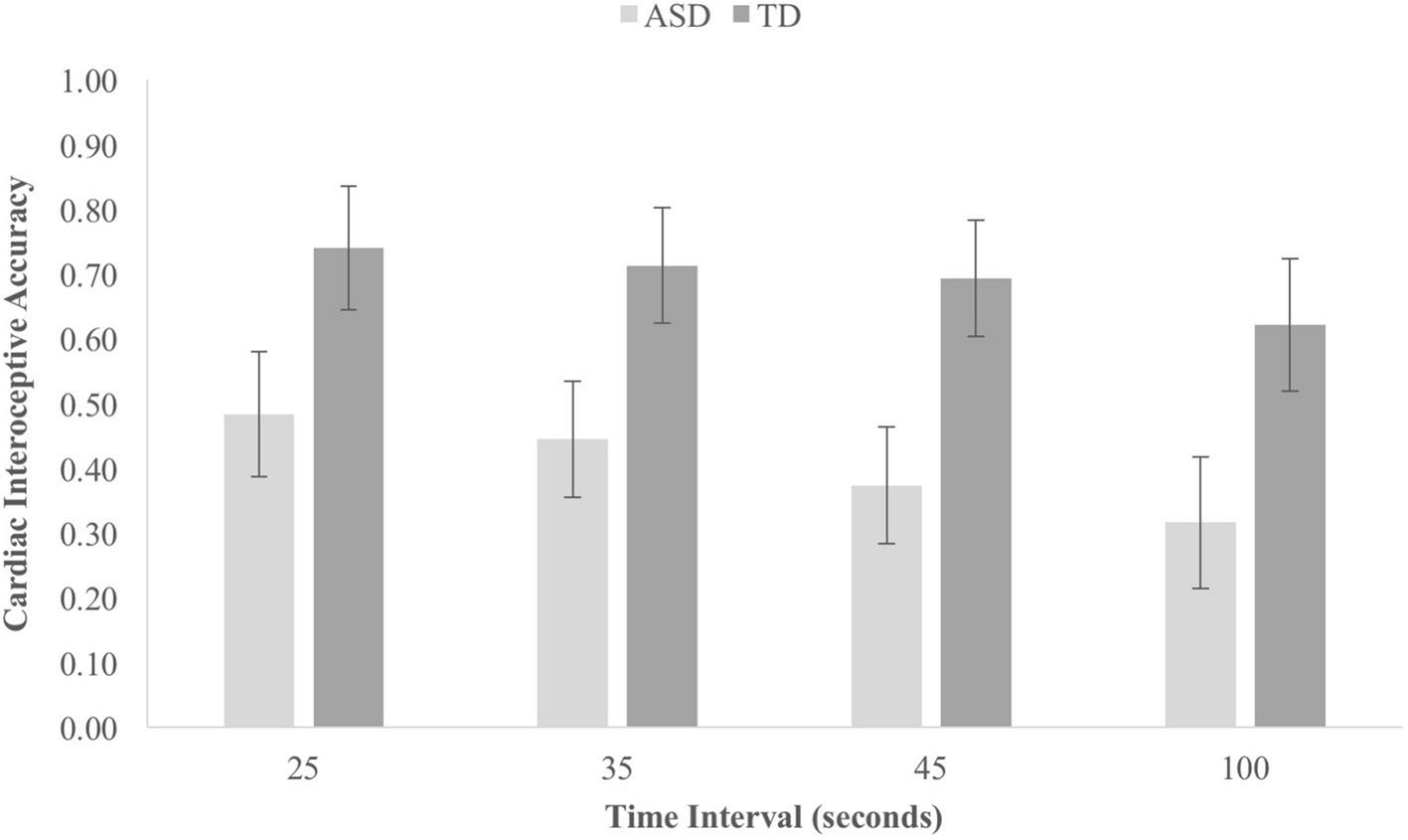
Cardiac interoceptive accuracy scores for each time period for each group during the HTT in experiment two. Error bars represent SEM

The average cIA score was 0.40 (SD 0.53) in the ASD group and 0.69 (SD 0.18) in the NT group, a difference which was moderate-to-large and statistically significant, *t*(40) = 2.34, *p* = 0.028, *d* = 0.72, BF^10^ = 2.51. The mean cIA score for each time interval on the heartbeat tracking task in each group is shown in Fig. 3. A 2 (group: ASD/NT) × 4 (time interval: 25 s/35 s/45 s/100 s) ANOVA was run on this data. The main effect of Group was significant, *F*(1,40) = 5.47, *p* = 0.024, $\eta_{p}^{2} = 0. 1 2$, but the Group × Time Interval interaction effect did not approach significance, *F*(3,120) = 0.25, *p* = 0.86, $\eta_{p}^{2} = 0.00 6$. Therefore, the ASD group showed diminished interoceptive accuracy across all time intervals, rather than with any particular duration.

#### Time Estimation Control

The average time estimation score was 0.81 (SD 0.15) in the ASD group and 0.88 (SD 0.10) in the NT group, a difference which was nonsignificant but which was moderate in magnitude, *t*(40) = 1.75, *p* = 0.09, *d* = 0.54, BF^10^ = 1.01. Crucially, however, performance on the time estimation task was non-significantly associated with the heartbeat tracking task in both the ASD group, *r* = 0.002, *p* = 0.99, BF^10^ = 0.27, and the NT group, *r* = 0.11, *p* = 0.60 BF^10^ = 0.30. Thus, while the NT group were significantly better at perceiving their heartbeat and numerically better at estimating time than the ASD group, the two measures failed to correlate significantly with each other within groups suggesting that the two measures were not tapping the same underlying process in either group, and that individuals were not merely counting during the heartbeat tracking task. The significant main effect of Group on heartbeat tracking task performance remained significant after controlling for time estimation performance, *F*(2,39) = 4.80, *p *= 0.03, $\eta_{p}^{2} = 0.11$. Indeed, the main effect of Group on heartbeat tracking task performance remained significant (with no change in the associated effect size) after controlling for all other background variables in addition to time estimation ability (age, VIQ, PIQ, and average heart rate), *F*(6,35) = 4.13, *p *= 0.05, $\eta_{p}^{2} = 0.11$.

#### Association Analyses

A series of bivariate correlations were conducted to explore the association between cIA, mindreading performance and SRS. Given that the results from association analyses were not a central focus the current study, combined with the fact that none of the key correlations was significant, we report the data in Online Supplementary Material.

### Experiment 2: Discussion

Interoceptive accuracy performance on the heartbeat tracking task was significantly lower in the ASD group than in the NT group, providing evidence that cIA is impaired in children with ASD. This is in line with a recent study by Palser et al. (2018) who also found a group of ASD children had diminished cIA on the same task compared to comparison participants. The current results could not be explained by other strategies such as counting, as there was no significant correlation between time estimation and performance on the heartbeat tracking task in either group. Moreover, even when controlling for time estimation performance their remained a significant difference in cIA between groups emphasising that time estimation ability could not explain the differences in performance on the heartbeat tracking task.

## General Discussion

Taken together, the results from experiments 1 and 2 add to the growing literature on interoception in ASD and provide a potential developmental explanation for how interoception changes across the lifespan in this condition. The findings partly support theories that reference the importance of altered interoceptive processing in the development of the ASD phenotype (Quattrocki and Friston 20 2014). However, the results also suggest that interoceptive difficulties in childhood may resolve by adulthood in people with ASD (unlike difficulties with mindreading and emotion processing, which remain impaired across the lifespan in ASD).

In Experiment 1, we replicated previous research findings of no cardiac interoceptive impairment in ASD adults (Shah et al. 2016; Mash et al. 2017; Nicholson et al. 2018), irrespective of the extent to which participants manifested alexithymic traits (cf. Nicholson et al. 2018). While one previous study (Garfinkel et al. 2016b) found an impairment in ASD adults, this study did not match groups for IQ, which leaves open the possibility that the finding was the result of between-group differences in general cognitive ability, rather than between-group differences in diagnostic status. While the current study found a clear medium-to-large cardiac interoceptive impairment in ASD children (*d *= 0.72), which is in line with another recent study by Palser et al. (2018), two other studies of cardiac interoception in children with ASD have observed no such impairment (Schauder et al. 2015; Mash et al. 2017). One issue to consider here is that, as highlighted by an anonymous reviewer of the manuscript, the average interoceptive accuracy of neurotypical children in Experiment 2 was higher than the average interoceptive accuracy of neurotypical adults in Experiment 1. While post hoc analysis showed that this difference was non-significant, *t *= 1.71, *p* = 0.10, *d* = 0.52, BF^10^ = 0.95, the fact that neurotypical children were even numerically superior to neurotypical adults is somewhat surprising. Perhaps these control children were unrepresentatively able, which produced artificial group differences in interoceptive accuracy that would not have been observed if “super controls” had not been employed. This is a possibility, but we guarded against this by ensuring the groups were matched for background variables, such as IQ and time estimation ability, which might have influenced interoceptive accuracy. The group difference in interoceptive accuracy remained significant after age, IQ, time estimation ability, and average heart rate were controlled in an ANCOVA. If the control children showed higher levels of interoceptive accuracy because they were super controls, then we might reasonably have expected to see group differences reduced or eliminated after controlling for other relevant variables, but this did not occur. When considering all these findings together, one interpretation is that there is heterogeneity in the cognitive profile of children with ASD and that cardiac interoception is impaired in only some children. However, whether such an impairment represents a core cognitive cause of ASD features is a matter for debate.

A number of theories have argued that emotional processing relies on inferring the causes of interoceptive signals (Wiens 2005; Seth 2013; Barrett 2017), and neuroimaging studies of the link between interoception and emotion provide some evidence in favour of this view (Critchley et al. 2004; Zaki et al. 2012). This fits when considering ASD, given that previous research has highlighted that difficulties in identifying and understanding emotions both in oneself and in others is a common cognitive-level difficulty in this disorder (Gaigg 2012; Uljarevic and Hamilton 2013; but see Williams and Happé 2010). If interoception is linked with emotion-processing development in neurotypical individuals, then early impairments interoception among children with ASD may underpin later (and persistent) difficulties with emotion-processing. In other words, it may be that a “decoupling” of interoception and emotion processing among some children with ASD results in emotions never being fully anchored within the body, making emotions difficult to understand in self and others across the lifespan even once interoception difficulties have resolved.

A related possibility concerning decoupling is that local interoceptive signals are not integrated together in a global sense, therefore restricting their influence on motivational and behavioural states which drive subsequent goal-directed action (Hatfield et al. 2019). This fits the pattern of weak central coherence often observed in people with ASD (Happé and Frith 2006), and suggests that interoceptive signals may be attended to by people with ASD in a more narrowly detail-focused manner than neurotypical people tend to attend to them. From this perspective the decoupling of interoceptive processing may not be specific to its links to emotional information per se, but global processing on a more general level, including the inability for interoceptive signals to inform emotional processing, but also that these processes struggle to properly bind with other information sources such as memory, perception and decision making. Future research aimed at measuring interoceptive, emotional and other forms of cognitive processing within the same population could test the plausibility of this account, while also shedding light on the idea that the decoupling may be specific to emotional and interoceptive processing.

Another alternative possibility is that the process of building theories of emotions (or mental states, generally) is impaired in ASD *independent* of interoception difficulties (e.g., Carruthers 2009; Williams 2010). This could also explain the findings that emotion-processing difficulties persist in ASD even after interoceptive difficulties have resolved, and also that emotion-processing abilities are not necessarily reliably associated with interoceptive accuracy (see Nicholson et al. 2018; also, the lack of a significant association in the current study). Future research should aim to unpick the developmental trajectories of both emotional and interoceptive processing in children with ASD, and investigate how these trajectories compare to those in neurotypical children.

The current results also speak to other theories such as the alexithymia hypothesis (Bird and Cook 2013), given that neither cardiac nor respiratory interoceptive accuracy was significantly associated with number of alexithymic traits in participants who completed Experiment 1. Moreover, there were no significant differences in interoceptive accuracy between individuals with ASD who scored over the clinical threshold for alexithymia (n = 13) and those who scored under threshold (n = 8). These findings challenge any strong claim that alexithymia and interoception are inextricably linked. A caveat here is that sample size was relatively modest and so drawing conclusions from the results of association (or subgroup) analyses should be done with caution. Nonetheless, the sample size is very similar to the sample size in previous studies that have reported an association between the two abilities (see Table 2). The alexithymia hypothesis is a plausible theory that explains some of the existing findings in the interoception-, emotion processing- and ASD-related literature. The current findings do not speak to all aspects of the theory and there are several aspects that may well be correct. The current findings suggest only that comorbid alexithymia does not appear to influence interoceptive abilities in adults with ASD as the theory predicts it should.

Aside from the contribution of the current results to our understanding of ASD, the results also have implications for theories of the structure of interoception. In particular, the finding that accuracy on the measure of cardiac interoception was not associated significantly with accuracy on the measure of respiratory interoception supports the suggestion that there may not be a unitary interoception faculty that processes all forms of interoceptive input (Garfinkel et al. 2016a). The finding that measures of interoceptive accuracy in different domains are not associated significantly does not show that the outputs from independent low-level interoceptive systems are not combined at a higher level of the processing hierarchy (e.g., at the level of integration or interpretation). However, it does imply that at the level of monitoring these systems are distinct. This can inform futures studies that aim to measure both interoceptive monitoring and integration across multiple domains.

In conclusion, the current study suggests that interoceptive accuracy impairments may be present in children with ASD, but that these impairments resolve over time and are absent by adulthood. This provides a developmental framework for understanding interoception in ASD and suggest future research should focus on how interoceptive and emotional processing relate to each other in children. Equally, it provides motivation for future studies aimed at improving interoception in childhood with the idea that this may have the potential to enhance the link between emotional processing and interoception early on, which could improve emotional processing in ASD through development and into adulthood. These findings add to the growing literature on interoception in ASD and provide insight into strategies for future research.

## Electronic supplementary material

Below is the link to the electronic supplementary material.

Supplementary material 1 (DOCX 33 kb)
