# Supplementary material for: Interoception is Impaired in Children, But Not Adults, with Autism Spectrum Disorder
Source: J Autism Dev Disord. 2019 May 24;49(9):3625–37. doi: 10.1007/s10803-019-04079-w (PMC6667420; doi:10.1007/s10803-019-04079-w)
Supplement: Supplementary file 1 — Supplementary material 1 (DOCX 33 kb) [file 10803_2019_4079_MOESM1_ESM.docx]

**Supplementary material**

**Experiment 1**

**Self-report measures.**

In addition to the experimental interoception tasks and mindreading tasks we also included a series of self-report measures in order to assess levels of depression, anxiety and alexithymia in our groups, as previous research has implicated them in interoceptive processing. Additionally we also calculated each participants BMI, another factor linked to changes in interoception, and collected a self-report measure of autistic traits to strengthen our investigation. Each of these measures is described below.

***Beck Depression Inventory 2 (BDI-II).***

The BDI-II (Beck, Steer, & Brown, 1996) is a reliable and widely used self-report questionnaire that consists of 21 aspects of depression (e.g. O'hara & Swain, 1996). In this questionnaire, individuals are asked to choose one statement for each aspect of depression that best describes their feelings the last two weeks including the day of the testing session (e.g. I am so sad or unhappy that I can’t stand it). Each statement is rated on a scale ranging from 0 to 3, with 0 being equivalent to no presence of the aspect and 3 indicating strong presence of the aspect. Scores range from 0 to 63, with higher scores indicating more severe depressive symptoms. A total score was obtained for each participant by summing all their responses.

***State-trait Anxiety Inventory (STAI).***

The STAI (Spielberger, Gorsuch, Lushene, Vagg, & Jacobs, 1983) is a reliable and widely used self-report questionnaire that measures state and trait anxiety (e.g. Bryant, Harvey, Dang, Sackville, & Basten, 1998). The STAI comprises 40 statements; half of them refer to current states, while the rest refer to general trait states. First, individuals are presented with statements such as “ I feel calm”, and they are asked to rate these statements, using a 4-point scale, ranging from “not at all” to “very much so”, based on their current feelings. These measure state anxiety. Then, individuals are presented with the statements that measure trait anxiety, such as “I am a steady person”, and they are asked to rate these statements using a 4-point scale, ranging from “almost never” to “almost always”, based on how they generally feel. Scores range from 40 to 160, with higher scores indicating greater levels of anxiety. We calculated an overall score of anxiety for each participant, by summing their state and trait anxiety subscores.

***Toronto alexithymia scale (TAS-20).***

The TAS-20 (Parker et al., 1994) is a reliable and widely used self-report questionnaire that assesses people’s ability to identify and describe feelings and emotions (e.g. Szatmari et al., 2008). It comprises 20 statements (e.g “I am able to describe my feelings easily”) with which individuals have to answer whether they completely disagree or completely agree on a 5-point scale. Scores ranges from 20 to 100. A score of ≥ 61 indicates clinically significant levels of alexithymia.

***Body mass index (BMI).***

Participants’ BMI was calculated using the following formula, BMI = (weight (kg) / height (cm))/height (cm).^3^

**Experiment 1 – Results**

**Bivariate correlations (Tables 1 & 2)**

cIA was nonsignificantly correlated with rIA in both the ASD group, *r* = .06, *p* = .80, and the NT group, *r* = .17, *p* = .46. None of the mindreading tasks were significantly associated with either cIA or rIA in either the ASD group (all *r*’s <.32, all *p*’s >.16) or the NT group (all *r*’s <.24, all *p*’s >.29). Likewise neither cIA or rIA were significantly associated with TAS20, BDI-II, STAI or BMI score in either the ASD group (all *r*’s <.25, all *p*’s >.27) or the NT group (all *r*’s <.29, all *p*’s >.21). Neither cIA or rIA were significantly associated with AQ score in either the ASD group (all *r*’s <.19, all *p*’s >.43) or the NT group (all *r*’s <.24, all *p*’s >.30).

**Partial correlations (Tables 3 & 4)**

In order to further assess the role of secondary measures partial correlations looked at the association between IA and AQ when controlling for secondary measures. The association between AQ and cIA remained nonsignificant when separately controlling for BMI, BDI-II & STAI in both the ASD group (all p’s> 0.44) and the NT group (all p’s> 0.20). Likewise, all associations between AQ and rIA remained nonsignificant when separately controlling for BMI, BDI-II & STAI in both the ASD group (all p’s> 0.33) and the NT group (all p’s> 0.37). Therefore, we found no evidence that secondary measures were driving our inability to find a statistically significant different in cardiac IA.

**Experiment 2 – Results**

**Bivariate correlations (Tables 5 & 6)**

cIA was nonsignificantly correlated with the both mindreading tasks, RMIE & Animations, as well as the Social responsiveness scale score in both the ASD group, all *r*’s < .27, all *p*’s >.23, and the NT group, all *r*’s < .28, all *p*’s >.21.

**^Bivariate correlations^**

**^Table A1^**

| **^ASD group (n=21)^** | ***^Measures^*** | **^rIA^** | **^AQ^** | **^RMIE^** | **^ANIM^** | **^TAS20^** | **^BDI-II^** | **^STAI^** | **^BMI^** | **^TE^** | **^MEM^** |
| --- | --- | --- | --- | --- | --- | --- | --- | --- | --- | --- | --- |
| ^Interoception^ | **^cIA^  *^r^***  ***^p^*** | ^.059^  ^.80^ | ^.092^  ^.69^ | ^.314^  ^.17^ | ^.288^  ^.21^ | ^.065^  ^.78^ | ^-.247^  ^.28^ | ^-.153^  ^.51^ | ^-.198^  ^.39^ | ^-.024^  ^.92^ | ^.089^  ^.70^ |
|  | **^rIA^ *^r^***  ***^p^*** |  | ^.182^  ^.43^ | ^.297^  ^.19^ | ^.275^  ^.23^ | ^.126^  ^.59^ | ^.113^  ^.63^ | ^.001^  ^.99^ | ^-.248^  ^.29^ | ^.178^  ^.44^ | ^.169^  ^.47^ |
| ^Autistic triats^ | **^AQ^  *^r^***  ***^p^*** |  |  | ^-.191^  ^.41^ | ^.295^  ^.19^ | ^.^**^644**^**  **^<.005^** | ^.300^  ^.19^ | ^.150^  ^.52^ | ^.082^  ^.73^ | ^.173^  ^.45^ | ^.192^  ^.40^ |
| ^Mindreading^ | **^RMIE^ *^r^***  ***^p^*** |  |  |  | **^.507^**  **^.02*^** | ^-.220^  ^.34^ | ^-.005^  ^.98^ | ^.098^  ^.67^ | **^-.445^**  **^.04*^** | ^.175^  ^.45^ | ^.121^  ^.60^ |
|  | **^ANIM^ *^r^***  ***^p^*** |  |  |  |  | ^.137^  ^.55^ | ^.212^  ^.36^ | ^.157^  ^.50^ | ^.036^  ^.88^ | ^.361^  ^.11^ | ^.084^  ^.72^ |
| ^Alexithymia^ | **^TAS20^ *^r^***  ***^p^*** |  |  |  |  |  | ^.307^  ^.18^ | ^.398^  ^.07^ | ^.329^  ^.15^ | ^.118^  ^.61^ | ^-.063^  ^.79^ |
| ^Depression^ | **^BDI-II^ *^r^***  ***^p^*** |  |  |  |  |  |  | **^.614**^**  **^<.005^** | ^.329^  ^.15^ | ^.429^  ^.05^ | ^-.239^  ^.30^ |
| ^Anxiety^ | **^STAI^ *^r^***  ***^p^*** |  |  |  |  |  |  |  | ^.220^  ^.34^ | ^.249^  ^.28^ | ^-.185^  ^.42^ |
| ^Weight^ | **^BMI^ *^r^***  ***^p^*** |  |  |  |  |  |  |  |  | ^.005^  ^.98^ | ^-.363^  ^.11^ |
| ^Control tasks^ | **^TE^ *^r^***  ***^p^*** |  |  |  |  |  |  |  |  |  | ^.179^  ^.44^ |

^cIA = cardiac Interoceptive Accuracy, rIA = respiratory Interoceptive Accuracy, AQ = Autism Quotient total score, RMIE = Reading the Mind in the Eyes proportion score, ANIM = Animations proportion score, TAS20 = Toronto Alexithymia Scale 20 score, BDI-II = Becks Depression Inventory 2 score, STAI = State-trait Anxiety Inventory score, BMI = Body mass index, TE = Time estimation score, MEM = Memory control score;^ *^r^* ^= Pearson Correlation,^ *^p^* ^= significance two-tailed. *^ *^p^* ^<.05, **^ *^p^* ^<.005.^

**^Table A2^**

| **^NT group (n=21)^** | ***^Measures^*** | **^rIA^** | **^AQ^** | **^RMIE^** | **^ANIM^** | **^TAS20^** | **^BDI-II^** | **^STAI^** | **^BMI^** | **^TE^** | **^MEM^** |
| --- | --- | --- | --- | --- | --- | --- | --- | --- | --- | --- | --- |
| ^Interoception^ | **^cIA^  *^r^***  ***^p^*** | ^.170^  ^.46^ | ^-.234^  ^.31^ | ^.078^  ^.74^ | ^-.121^  ^.60^ | ^.283^  ^.22^ | ^-.149^  ^.52^ | ^-.129^  ^.58^ | ^.124^  ^.59^ | ^.187^  ^.42^ | ^.420^  ^.07^ |
|  | **^rIA^ *^r^***  ***^p^*** |  | ^.095^  ^.68^ | ^.239^  ^.30^ | ^-.014^  ^.95^ | ^.278^  ^.22^ | ^.121^  ^.60^ | ^.205^  ^.37^ | ^-.279^  ^.22^ | ^.197^  ^.39^ | ^.233^  ^.322^ |
| ^Autistic traits^ | **^AQ^  *^r^***  ***^p^*** |  |  | ^-.281^  ^.22^ | ^.088^  ^.70^ | **^.522*^**  **^<.05^** | **^.731***^**  **^<.001^** | ^.417^  ^.06^ | ^.337^  ^.14^ | ^.224^  ^.33^ | ^-.292^  ^.21^ |
| ^Mindreading^ | **^RMIE^ *^r^***  ***^p^*** |  |  |  | ^-.097^  ^.68^ | ^-.366^  ^.10^ | ^-.058^  ^.80^ | ^-.022^  ^.92^ | ^-.035^  ^.88^ | ^.065^  ^.78^ | ^-.050^  ^.84^ |
|  | **^ANIM^ *^r^***  ***^p^*** |  |  |  |  | ^.279^  ^.22^ | ^-.051^  ^.83^ | ^.278^  ^.22^ | ^-.019^  ^.94^ | ^-.013^  ^.96^ | ^-.129^  ^.59^ |
| ^Alexithymia^ | **^TAS20^ *^r^***  ***^p^*** |  |  |  |  |  | **^.531*^**  **^<.05^** | **^.581*^**  **^<.05^** | ^-.028^  ^.90^ | ^.162^  ^.48^ | ^.133^  ^.58^ |
| ^Depression^ | **^BDI-II^ *^r^***  ***^p^*** |  |  |  |  |  |  | **^.717***^**  **^<.001^** | ^.051^  ^.83^ | ^-.001^  ^.99^ | ^-.214^  ^.37^ |
| ^Anxiety^ | **^STAI^ *^r^***  ***^p^*** |  |  |  |  |  |  |  | ^-.238^  ^.30^ | ^.051^  ^.83^ | ^.025^  ^.92^ |
| ^Weight^ | **^BMI^ *^r^***  ***^p^*** |  |  |  |  |  |  |  |  | ^.032^  ^.89^ | ^-.308^  ^.19^ |
| ^Control tasks^ | **^TE^ *^r^***  ***^p^*** |  |  |  |  |  |  |  |  |  | ^.226^  ^.34^ |

^cIA = cardiac Interoceptive Accuracy, rIA = respiratory Interoceptive Accuracy, AQ = Autism Quotient total score, RMIE = Reading the Mind in the Eyes proportion score, ANIM = Animations proportion score, TAS20 = Toronto Alexithymia Scale 20 score, BDI-II = Becks Depression Inventory 2 score, STAI = State-trait Anxiety Inventory score, BMI = Body mass index, TE = Time estimation score, MEM = Memory control score;^ *^r^* ^= Pearson Correlation,^ *^p^* ^= significance two-tailed. *^ *^p^* ^<.05, **^ *^p^* ^<.005,***^ *^p^* ^<.001.^

**^Partial Correlations^**

**^Table A3^**

| **^ASD (n=21)^**  ^Control variables^ |  | **^cIA^** | **^rIA^** |
| --- | --- | --- | --- |
| **^BMI^** | ***^r^***  **^AQ^ *^p^*** | ^.110^  ^.64^ | ^.209^  ^.38^ |
| **^BDI-II^** | ***^r^***  **^AQ^ *^p^*** | ^.179^  ^.45^ | ^.156^  ^.51^ |
| **^STAI^** | ***^r^***  **^AQ^ *^p^*** | ^.117^  ^.62^ | ^.184^  ^.44^ |

^cIA = cardiac Interoceptive Accuracy, rIA = respiratory Interoceptive Accuracy, AQ = Autism Quotient total score, BDI-II = Becks Depression Inventory 2 score, STAI = State-trait Anxiety Inventory score, BMI = Body mass index; *^ *^p^* ^<.05, **^ *^p^* ^<.005,***^ *^p^* ^<.001.^

**^Table A4^**

| **^NT (n=21)^**  ^Control variables^ |  | **^cIA^** | **^rIA^** |
| --- | --- | --- | --- |
| **^BMI^** | ***^r^***  **^AQ^ *^p^*** | ^-.295^  ^.21^ | ^.209^  ^.38^ |
| **^BDI-II^** | ***^r^***  **^AQ^ *^p^*** | ^-.185^  ^.43^ | ^.010^  ^.97^ |
| **^STAI^** | ***^r^***  **^AQ^ *^p^*** | ^-.200^  ^.40^ | ^.011^  ^.97^ |

^cIA = cardiac Interoceptive Accuracy, rIA = respiratory Interoceptive Accuracy, AQ = Autism Quotient total score, BDI-II = Becks Depression Inventory 2 score, STAI = State-trait Anxiety Inventory score, BMI = Body mass index; *^ *^p^* ^<.05, **^ *^p^* ^<.005,***^ *^p^* ^<.001.^

**^Experiment 2^**

**^Table A5^**

| **^ASD group (n=21)^** | ***^Measures^*** | **^SRS^** | **^RMIE^**  **^(n=20)^** | **^ANIM^** | **^TE^** |
| --- | --- | --- | --- | --- | --- |
| ^Interoception^ | **^cIA^ *^r^***  ***^p^*** | ^-.269^  ^.24^ | ^.128^  ^.59^ | ^.080^  ^.73^ | ^.002^  ^.99^ |
| ^Autistic traits^ | **^SRS^  *^r^***  ***^p^*** |  | ^-.314^  ^.18^ | ^.310^  ^.17^ | ^-.215^  ^.35^ |
| ^Mindreading^ | **^RMIE^ *^r^***  **^(n=20)^ *^p^*** |  |  | ^.019^  ^.94^ | ^-.095^  ^.69^ |
|  | **^ANIM^ *^r^***  ***^p^*** |  |  |  | **^-.587*^**  **^<.05^** |

^cIA = cardiac Interoceptive Accuracy, SRS= Social Responsiveness Scale T score, RMIE = Reading the Mind in the Eyes proportion score, ANIM = Animations proportion score, TE = Time estimation score;^ *^r^* ^= Pearson Correlation,^ *^p^* ^= significance two-tailed. *^ *^p^* ^<.05, **^ *^p^* ^<.005,***^ *^p^* ^<.001.^

**^Table A6^**

| **^NT group (n=21)^** | ***^Measures^*** | **^SRS^** | **^RMIE^** | **^ANIM^** | **^TE^** |
| --- | --- | --- | --- | --- | --- |
| ^Interoception^ | **^cIA^ *^r^***  ***^p^*** | ^.274^  ^.23^ | ^-.014^  ^.95^ | ^-.278^  ^.22^ | ^.111^  ^.63^ |
| ^Autistic traits^ | **^SRS^  *^r^***  ***^p^*** |  | **^-.490*^**  **^<.05^** | **^-.581*^**  **^<.05^** | ^.155^  ^.50^ |
| ^Mindreading^ | **^RMIE^ *^r^***  ***^p^*** |  |  | ^.004^  ^.99^ | ^.240^  ^.30^ |
|  | **^ANIM^ *^r^***  ***^p^*** |  |  |  | **^-.531*^**  **^<.05^** |

^cIA = cardiac Interoceptive Accuracy, SRS= Social Responsiveness Scale T score, RMIE = Reading the Mind in the Eyes proportion score, ANIM = Animations proportion score, TE = Time estimation score;^ *^r^* ^= Pearson Correlation,^ *^p^* ^= significance two-tailed. *^ *^p^* ^<.05, **^ *^p^* ^<.005,***^ *^p^* ^<.001.^
